# Development of a mobile-based intelligent module for identification and tracking of household appliances

**DOI:** 10.1038/s41598-023-42656-3

**Published:** 2023-10-05

**Authors:** Dmitrii Kaplun, Irina Shpakovskaya, Aleksandr Sinitca, George Efimenko, Malak S. Alqahtani, Rania M. Ghoniem, Mohamed Abbas, Ben Othman Soufiene, Sergey Romanov

**Affiliations:** 1 Artificial Intelligence Research Institute, China University of Mining and Technology, 221116 Xuzhou, China; 2 Department of Automation and Control Processes, Saint Petersburg Electrotechnical University “LETI”, Saint Petersburg, Russia 197022; 3 Centre for Digital Telecommunication Technologies, Saint Petersburg Electrotechnical University “LETI”, Saint Petersburg, Russia 197022; 4 Computer Engineering Department, College of Computer Science, King Khalid University, 61421 Abha, Saudi Arabia; 5 Department of Information Technology, College of Computer and Information Sciences, Princess Nourah bint Abdulrahman University, P.O. Box 84428, 11671 Riyadh, Saudi Arabia; 6 Electrical Engineering Department, College of Engineering, King Khalid University, 61421 Abha, Saudi Arabia; 7 PRINCE Laboratory Research, ISITcom, Hammam Sousse, University of Sousse, Sousse, Tunisia

**Keywords:** Energy science and technology, Engineering

## Abstract

Every year manufacturers of household appliances improve their devices, trying to make everyday life easier for users. New smart devices have many useful features, but not all users can easily cope with the complexity of the devices. One of the main tasks of household appliance manufacturers is to ensure the convenience of using appliances, taking into account the increasing complexity. Therefore, any manufacturer supplies equipment with a short but useful instruction manual. Practice shows that no printed user manual can compare with a demonstration of the device operation by a professional consultant. Instructions for home appliances using augmented reality technology will allow users to get the necessary detailed information about the device in a short period of time. As part of this work, the task of developing an artificial intelligence-based module is being solved. This module consists of developed classification, matching, and tracking submodules that can provide simple and fast visual instructions to users of household appliances in real time. The identification of household appliances is performed with more than 0.9 accuracy, and the tracking inside an unidentified object using the camera of a mobile device is processed with the success score of about 0.68 and frames per second (FPS) about 7. Mobile applications based on the proposed intelligent modules for Android and iOS were developed.

## Introduction

Manufacturers of household appliances are constantly improving their devices, trying to make everyday life easier for users. New "smart" devices have many useful features, but not all users can easily cope with the complexity of devices, which grows exponentially.

Classic user manuals for household appliances tend to be large and inconvenient. Such actions as: unboxing and installation, feature introduction, and troubleshooting are difficult to describe in classic instructions.

The use of modern technologies such as augmented reality to present user instructions can speed up and simplify the user’s work with household appliances. The use of modern technology in the development of user instructions also improves the following points:

- Lower service center load (are less calls to customer service asking for help).
- Reducing home calls for troubleshooting (is a reduced need to dispatch technicians to customers’ homes).
- Reducing the number of returns of equipment due to the complexity or impossibility of using.

To provide access to the user’s manual, it is necessary to determine the position of the interface elements in the frame at each moment of time. Directly, this task is the task of tracking. However, first it is necessary to determine which model of household appliances is in the frame.

To determine the type and model of the household appliance, it is possible to apply well-developed classifiers based on neural networks. The main problem of such solutions is that there are some problems related to dataset limitations: (1) not all models are represented in the dataset. It is often difficult to collect training samples to exhaust all classes when training a recognizer or classifier. This problem is related to the Open Set Recognition (OSR) problem, where incomplete knowledge of the world exists at training time, and unknown classes can be submitted to the algorithm during testing, requiring the classifiers to not only accurately classify the seen classes, but also to effectively deal with unseen ones^1^. (2) Little data for each model. Problems where it is necessary to classify models on data for which there are only a few training samples with labeled information refer to Few-Shot Learning (Few-Shot Learning, i.e., classifying new data when you have only a few training samples with supervised information)^2^.

There are various approaches to solving the OSR problem. For example, using data only from the classes under consideration or assuming the presence of some dataset of negative examples. A detailed overview of the methods used to solve the problem is presented in^1^.

At the time of the prototype development, the restriction was accepted that the user, by default, points the camera at the type of household appliances that are in the dataset.

In this work, to solve the problem of data limitation for each class, metric-based few-shot classification methods were applied^3^.

After classifying by type and by model of household appliances, it is necessary to determine which key points (interface buttons) need to be tracked. It is possible to solve this problem as a detection problem, but this requires a larger training sample. In this case, the mutual locations of key points will not be calculated, and it is also computationally expensive to add a new class of household appliances.

In this work, a different approach is chosen, matching a template on which the interface elements are marked. Inserting a new class of household appliances will be carried out quickly. Requires a definition on the photograph of the coordinates of the interface elements.

Recently, the match was based on SuperPoint + SuperGlue^4,5^. To search and match key points, a bundle of pre-trained SuperPoint and SuperGlue networks is used in our work. The advantage of this approach is that there is no need to retrain the network when adding new models or types of household appliances.

The result of determining the object class and key interface points is fed to the input of the tracker module. Recently, deep neural network-based object tracking algorithms have emerged and have obtained great attention from researchers due to their outstanding tracking performance^6^. Some of the most popular tracking methods are: DeepSORT^7^, ROLO^8^, SiamMask^9^, JDE model^10^, Tracktor++^11^. In the work^12^ SiamFC++ proves both the tracking and generalization abilities of the tracker. The SiamFC++ tracker was shown to achieve maximum performance in different applications. In this work, a fully convolutional Siamese tracker++ (SiamFC++) was used that was trained on the GOT-10k dataset^12^.

There are several augmented reality solutions for user manuals on the market. MobiDev company^13^, Quytech company^14^ are developing tools to create instructions with augmented virtual reality for industry and household appliances, but these solutions for tracking implementation on iOS and Android-based mobile platforms use ARKit^15^ and ARCore^15^ libraries. There are some problems with using these libraries. First, the barrier to using these libraries is that they are new at the time of development—only supported on newer devices. Second, these libraries are not allowed to be modified, so it is impossible to do anything with them from the point of improvement of quality or computational complexity. Moreover, the Quytech company tool doesn’t provide models recognition.

Some solutions were described in the papers. The paper^16^ describes the GuideMe mobile application, which provides users with interactive instructions for home appliances based on augmented reality. There is no information about types or models recognition in this paper. The paper^17^ also solves similar problems—Object Detection, Point Cloud Registration, Initial Pose Estimation. The proposed algorithm requires knowledge of a 3D model of the object and an initial estimation of the object pose based on deep learning-based detection of proper object features, in order to improve the accuracy and speed of the registration process. It can be a problem because manufacturers don’t always provide 3D models of their household appliances.

Although there are some solutions, the major appliance manufacturers are limited to standard instructions. This leads to the assumption that existing solutions do not have sufficient quality and ease of deployment. Moreover, all existing news applications work with a limited set of appliances and have problems with adding new models, speed, and quality of work. Therefore, when developing the mobile-based application, the following requirements should be set:

- ease of adding of a new model of household appliances;
- speed of recognition of household appliances;
- classification accuracy;
- tracking speed.

Finally, the overview and main contributions of our work can be summarized as follows:

- Development of a neural network training algorithm based on visual data.
- Optimize the neural network model to ensure performance and reduce the number of incorrect recognitions.
- Development of an algorithm for retraining the neural network model based on the data collected by the application about objects.
- An AI module has been developed that allows for the classification of household appliances objects “scanned” using the camera of a mobile device.
- Development and efficient implementation on mobile devices a space tracking algorithm for accurate positioning of visual objects relative to real world objects.

The paper is organized as follows. In Section "Materials and methods", we present the pipeline of our approach and comment on its stages. In Section "Implementation on mobile devices", we describe the implementation of the proposed approach on mobile platforms. The numerical results and discussion are presented in Sections "Results" and "Discussion". Finally, Section "Conclusions" reports on the conclusions and future works.

## Materials and methods

**Figure 1:**
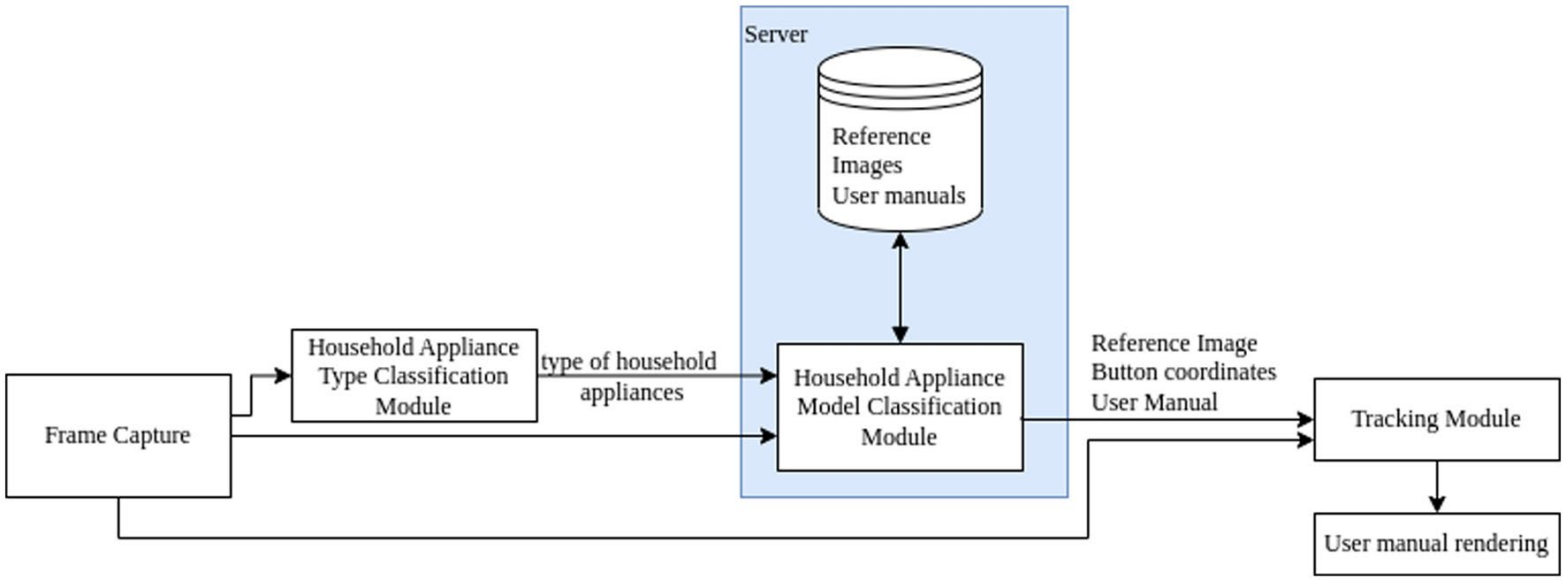
Flowchart of the proposed solution (the modules highlighted with blue rectangle shall be executed on the remote server side, other modules are executed locally on the mobile device).

A flowchart of the developed solution is presented in Fig. 1. As shown, the first step is frame acquisition from the device camera. Next, on the device side, a household appliance classification is performed, resulting in a grouping with an image sent to the remote server. The next step is executed on the cloud server, namely, model classification. Based on the determined model, the server returns the following information to the device: reference image, button coordinates, and user manual. Finally, the video stream from the device camera and the information from the server are routed to the tracking module, which provides information for the user manual rendering.

It can be seen that the main modules performed on the mobile device are the classification and tracking modules. The tracking module consists of the matching and tracking submodules. Based on the above, we need to describe:

1. Classification module.
2. Matching submodule.
3. Tracking submodule.

### Classification module

**Figure 2:**
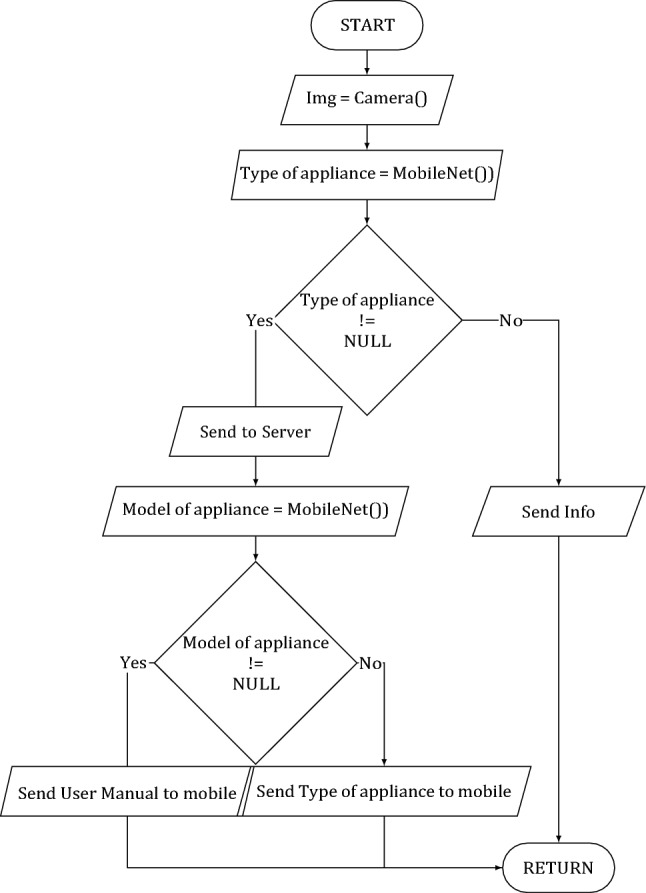
Classification module flowchart.

A flowchart of the classification module is presented in Fig. 2.

At the stage of classification it is necessary to solve two problems:

- Determination of the type of household appliances.
- Determining the model of household appliances.

When video is shot on a mobile device, the image is captured and sent to the classification module by type of household appliances. The result of the work of the classification module by type of household appliances is the probability vector of household appliances belonging to each of the given classes. The probability vector and the image are then sent to the server. The image is sent to the input of the classification model to determine the technique model.

This classifier architecture was chosen because it was supposed to add new models of household appliances. If the model was deployed to a mobile phone, then when a new model of household appliances was added, an update of the mobile application was required. Another reason to deploy the home appliance detection model on the server is to increase the network in the future, using a more heavyweight but accurate solution.

One of the required features of the developed system was the possibility to classify image as ”nothing”. However, the open-set classification approach can be a potential bottleneck for pipeline performance, and as the classificator is used the MobileNet^18^ network based detector. MobileNet uses depthwise separable convolutions. It significantly reduces the number of parameters when compared to the network with regular convolutions with the same depth in the net.

### Matching submodule

**Figure 3:**
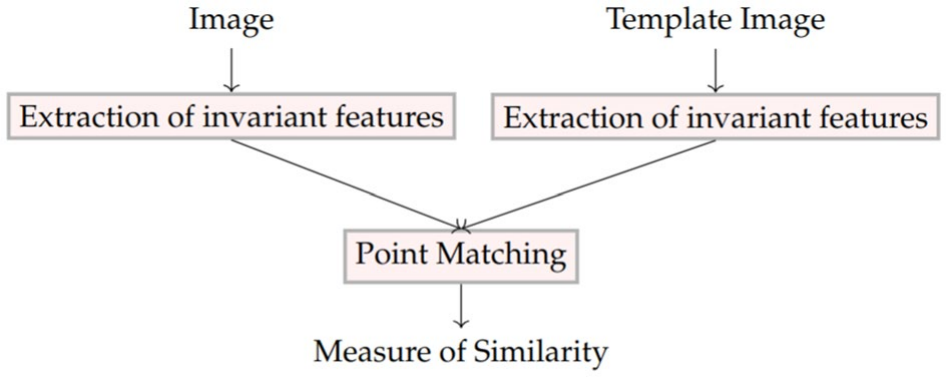
Matching submodule structure.

**Figure 4:**
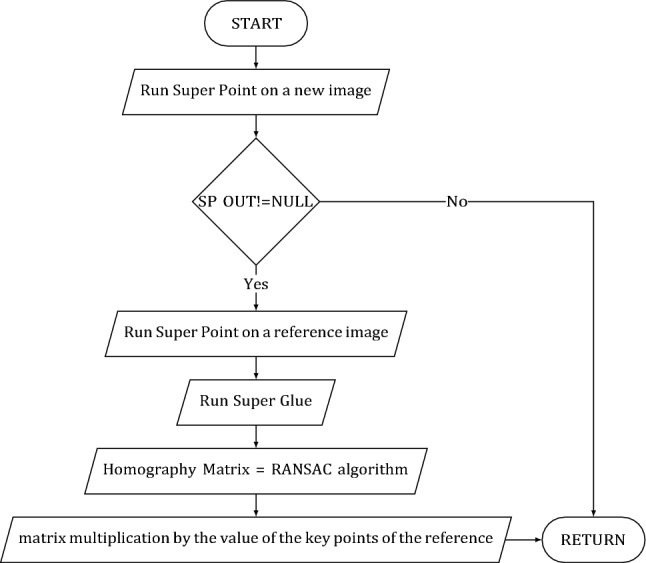
Matching submodule flowchart.

The structure of the matching submodule and its flowchart are presented in Figs. 3 and 4, respectively.

In this step, the key points of the image are detected and compared. Point-matching task is a classic computer vision problem. Until recently, classical methods (SIFT, SURF, SIRF, ORB, and others) were more often used to solve this problem; at the moment, neural networks are better able to cope with this task. Classical methods are inferior to learning-based methods, as they are difficult to properly tune the parameters^19^.

To detection and compare key points, a bundle of pre-trained SuperPoint and Super-Glue networks are used. The advantage of this approach is that there is no need to retrainthe network when adding new models or types of equipment.

A fully convolutional neural network SuperPoint first pretrains an interest points detector on synthetic data and then learns the descriptors by generating image pairs with known homography transformation.

SuperPoint is a self-supervised framework for training interest point detectors. It first pretrains an interest point detector on synthetic data and then learns the descriptors by generating image pairs with known homography transformation^4^.

Note that SuperPoint lasts significantly longer than SuperGlue. In this paper, to speed up the SuperPoint network can be divided into two parts. The first part can run on the GPU, and the second on the CPU. What will be discussed next.

The SuperGlue graph neural network^5^ is used in the work to find matches and reject incompatible points. It shows the best on the control data sets. This model receives keypoints and two image descriptors as input, which can be obtained by any method. However, the best quality is achieved when using the SuperPoint network for this purpose. SuperGlue, unlike other approaches, seeks to perform context aggregation, matching, and filtering in a single end-to-end architecture. There are two main components in the SuperGlue architecture: Attentional Graph Neural Network and Optimal Matching Layer.

SuperGlue uses two kinds of attention: self-attention to boost the receptive field of local descriptors and cross-attention to enable cross-image communication and is inspired by the way humans compare photos (by looking back-and-forth).

The advantage of using a bundle of SuperPoint and SuperGlue networks is that there is no need to retrain the network when adding new models or types of household appliances.

Next, we obtain the coordinates of the interface details of interest on the input image. For this, a homography matrix is constructed.

In computer vision, two images of the same object in space are connected by homography. So, having a set of points on the reference image and a set of points in the scene associated with it, we can find a correspondence between them in the form of a homography matrix H. The RANSAC^20^ algorithm was used to find this transformation. The algorithm evaluates homography for randomly selected points and does so until a sufficient match between coordinates is achieved. The advantage of the RANSAC algorithm is its ability to give a reliable estimate of the model parameters, i.e., the ability to estimate model parameters with high accuracy, even if there is a significant amount of outliers in the original dataset. After calculating the homography matrix, a perspective matrix transformation of vectors is performed, i.e., multiplication of the homography matrix by the coordinates of points on the reference image. This operation allows you to find the desired coordinates on the frame. Obtaining the homography matrix and perspective transformation of coordinates is performed using the functions of the OpenCV library.

**Figure 5:**
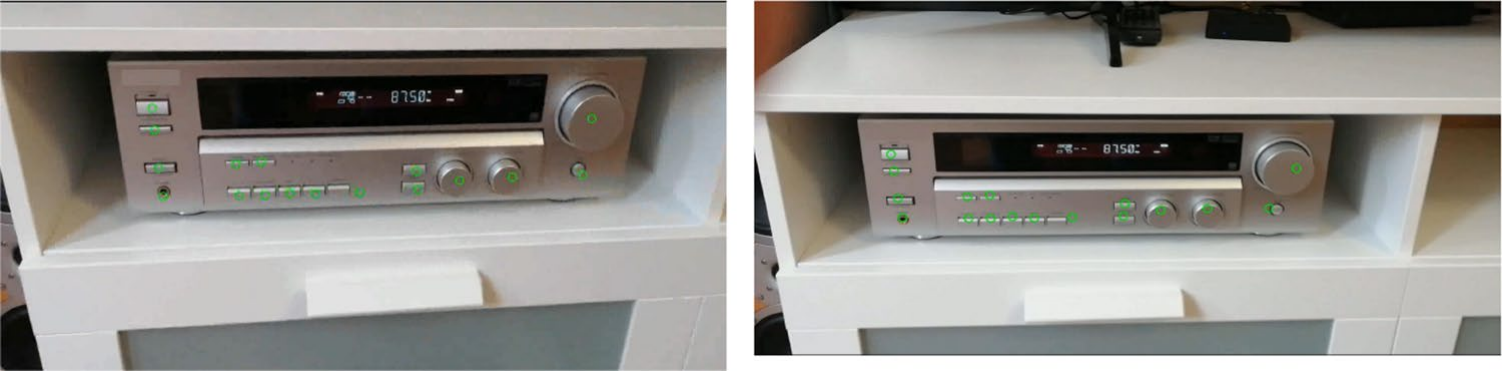
Examples of the developed matching submodule work for audio video surround receiver.

Examples of the developed matching submodule work are presented in Fig. 5 with the use of the audio video surround receiver.

### Tracking submodule

The tracking problem presented in this paper, depending on the amount of tracking, belongs to the single object tracking (SOT) tracking class. It seems that the task is Multi-Object Tracking (MOT) or Multi-Target Tracking (MTT), but as the tracking objects will not move relative to each other, the task belongs to SOT^21^.

The tracking component must be able to track previously unknown and quite diverse objects. For these purposes, general-purpose trackers that do not specialize in any objects are better suited. There are a large number of datasets for training and testing general-purpose trackers, such as LaSOT, VOT, TrackingNet, GOT-10k and others. Thus, for the task under consideration, a tracker is suitable, which, on the one hand, would give the maximum speed on the indicated and other datasets, on the other hand, would be fast enough to provide the necessary performance requirements. As a result of the analysis, the SiamFC++ tracker^22^ and its implementation in the Video Analyst library^23^ were chosen.

The tracker model consists of four convolution layers, a basemodel backbone, and a head. There are several models prepared with different backbones^24^. The siamfcpp-shufflenetv2 × 0_5 model, trained on the GOT-10k dataset, was taken into account in the work, since it provides maximum performance.

The challenging problem was to implement tracking from different sides. The algorithm (Algorithm 1) was proposed to solve this problem.
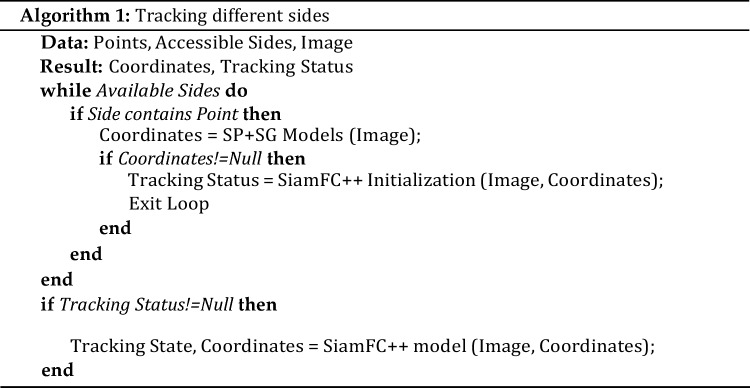


**Figure 6:**
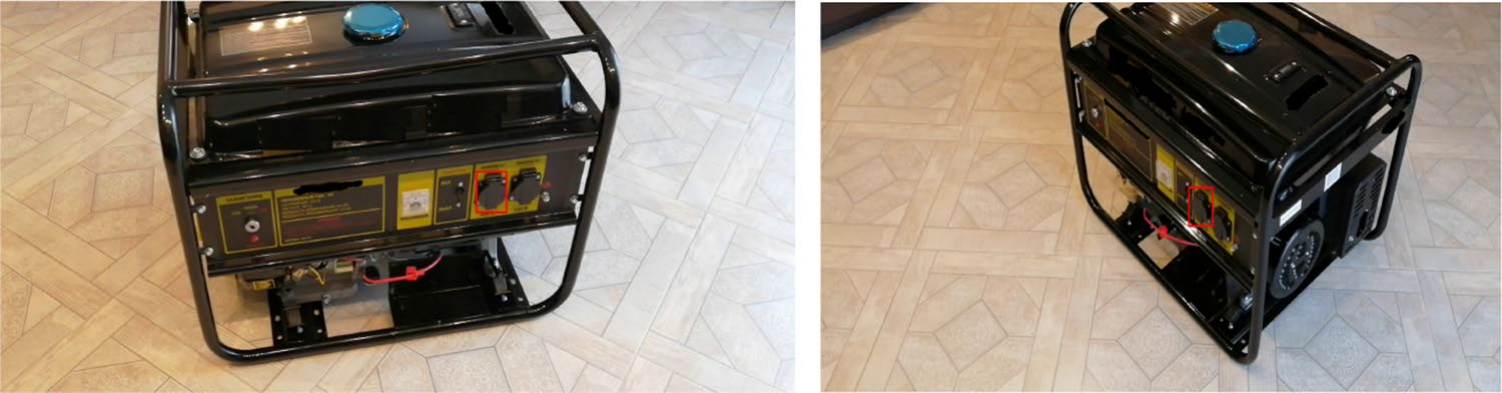
Examples of the developed tracking module work for generator.

Examples of the developed tracking module work are presented in Fig. 6 for the generator.

All modules, their architecture, and implementation are presented in Table 1.

**Table 1:** Modules implementation.

Modules	Architecture	Processor	Location
Classification by type	MobileNet V2	GPU	In the mobile
Classification by model	MobileNet V2	GPU	On the server
Matching	SuperPoint	GPU	In the mobile
		CPU	
	SuperGlue	CPU	
Tracking	SiamFC++	CPU	

### Dataset description

In our work, for the classification of types and models, we used the following dataset consisting of 1480 images divided into six classes. The division by type of equipment is presented in the Table. 2.

**Table 2:** Dataset division by type of equipment.

Class	Number of images
Oven	90
Coffeemakers (coffee machines)	15
Microwave ovens	110
Multicookers	70
Dishwashers	95
Washing machines	1100

When training the classification model according to the household appliance models, 160 actual household appliance models were used. The dataset looks strongly imbalanced, but this problem was solved by using different augmentation techniques such as RandomHorizontalFlip, Cutout, etc.

These datasets were collected mainly on hypermarket appliances during daylight hours. The images were taken with a phone camera with a resolution of 960 by 1280 pixels in JPEG format. Additionally, data were used from open datasets: Caltech Home Objects 2006^25^, and Office-Home Dataset^26^.

351 images were used to test the model to determine the type of household appliances, and 370 images were used to test the model to determine the model of household appliances. The data was splitted into train/test in the percentage ratio of 80/20 for each class.

## Implementation on mobile devices

One of the main tasks of the project is the deployment of modules on mobile devices. Currently, there are critical problems that complicate the process of inference and training neural networks on mobile devices. Of these, three main ones are distinguished, which come from hardware characteristics:

- Limited supply of energy.
- Limited memory space;
- Low computing power.

During implementation, it is important to maintain a balance between the accuracy of the model predictions and the computational resources used. Therefore, another important task is the reducing the size of the network, optimization algorithms are used: pruning, quantization. Several algorithms for compressing and accelerating the models are commonly used for this. The use of such algorithms affords speed up model inference and reduces model size without reducing the quality of network prediction.

In general, techniques for compacting and DNN models are divided into four categories: parameter pruning and quantization, low-rank factorization, transferred/compact convolutional filters, and knowledge distillation^27,28^.

After theoretical and experimental research, a post-training static quantization algorithm was chosen for solving these problems. This method provides a reduction in hardware and software costs, as well as ease of implementation: the method is implemented in the PyTorch library.

The idea behind quantization is that converting weights and inputs to integer types results in less memory consumption and faster calculations on certain hardware. Pytorch implements several quantization modes:

- Post-Training Dynamic – the weights are quantized ahead of time but the activations are dynamically quantized during inference.
- Post-Training Static Quantization – pre-quantizes model weights and the clipping range is pre-calibrated and fixed (“static”) using validation data.
- Quantization-Aware Training models the effects of quantization during training.

After models are trained and quantized, one essential step before the model can be used in iOS and Android apps is to convert the Python-dependent model to TorchScript, which can then be further optimized for mobile apps.

However, deploying and running a custom neural network on a phone or tablet is not straightforward, and the process depends on the operating system of the machine. Conversion to each of the platforms has its own specifics. For example, one of the most convenient ways to deploy a network on the iOS platform:

1. Export in Open Neural Network Exchange (ONNX) format^29^.
2. Conversion to CoreML for iOS platforms^30^.

But it is not possible to convert it to the CoreML format, because the object tracking solution presented in the work has a non-standard model architecture.

One of the options to implement the tracking module on the iOS and Android-based mobile platforms is the use of ARKit and ARCore. However, there are some problems with using these libraries. First, the barrier to using these libraries is that they are new at the time of development—only supported on newer devices. Second, these libraries are not allowed to be modified, so it is impossible to do anything with them from the point of improvement of quality or computational complexity.

As a result of serialization, a model will be synthesized in TorchScript (.pt) format. TorchScript model can then be run in a high-performance environment. TorchScript supports GPU calculations.

**Figure 7:**
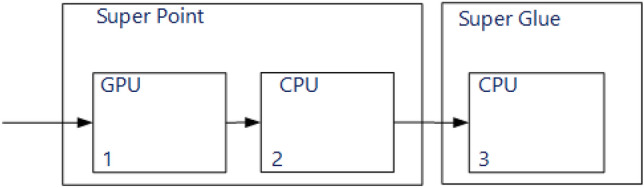
Functional diagram of the developed solution.

But in the work it was not possible to convert the entire SuperPoint on the GPU, because for some functions (e.g. from Numpy lib) such conversion is not supported in the version of PyTorch 1.6.0 (CUDA 10.1). In the SuperPoint algorithm, the neural network is first output, then the output results are post-processed, which is more expedient to do on the CPU. Therefore, the SuperPoint algorithm was divided into two parts: neural network output (performed on the GPU), post-processing of the output results (performed on the CPU); see Table 1 and Fig. 7.

The module interface is implemented in native OS programming languages: SWIFT and Java on iOS and Android accordingly.

**Figure 8:**
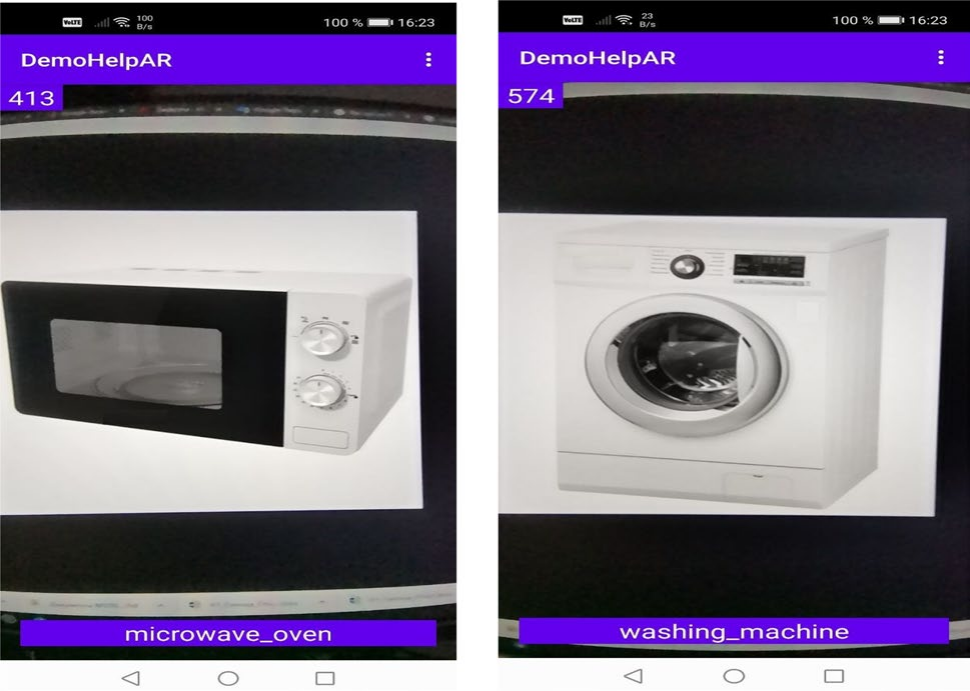
Examples of how classification works on the Android mobile platform for microwave oven and washing machine.

Examples of the developed classification module work on a mobile platform are presented in Fig. 8 for the microwave oven and washing machine.

**Figure 9:**
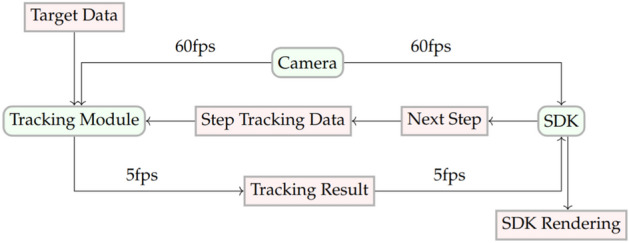
Timing diagram of the interaction between the SDK and the neural network module for tracking.

As soon as the module receives classification data, it gives it to the SDK—this is the software part of the mobile application, which includes working with the network, the camera manager and rendering the image, asynchronously via a callback or a delegate method (Fig. 9).

Table 3 describes the inputs and outputs of the main tracking components. The function opens a grayscale image with pixel values ranging from 0 to 1 and results in a dimension of (1, 1, resize[0], resize[1]). The resize argument is a list of the width and height to which the image is resized. Two variants of the width and height values were considered: [640, 480] for better quality or [320, 240] for faster performance.

**Table 3:** Input–output tracking submodules.

Part of model	Input	Output
sp_firts_part.pt	Transformed image	Intermediate scores, intermediate descriptors
sp_cpu_part_pytorch.pt	Descriptors, scores	Keypoints, descriptors, scores
superglue_torchscript.pt	Descriptors_template, descriptors_frame, keypoints_template, keypoints_frame, scores_template, scores_frame	Indexes of corresponding keypoints in another image: indices0, indices1, mscores0, mscores1
tracker_init.pt	Frame, init_box—the coordinates of the object to be tracked	State—the state for the entry tracker_update; features, windows
tracker_update.pt	Frame, state, features, window	rect_pred—coordinates of the estimated box, state, pscore—tracker score

After that, the transformed image is fed to the input of the *sp_firts_part.pt* model. The output returns scores and descriptors tensors of dimensions (1, 65, x, y) and (1, 256, x, y), respectively, where x = 60, y = 80 with resize = [640, 480] and x = 30, y = 40 with resize = [320, 240]. Next, the scores and descriptors tensors are fed to the *sp_cpu_part_pytorch.pt* model. The keypoints, descriptors, scores tensors of dimensions [N, 2], [256, N] and [N] are returned to the output, respectively, where N is the number of key points found, which is not known in advance and may vary depending on the input image and/or parameters.

The reference image and the current frame are fed to the SuperPoint input. So, there are two outputs: *descriptors_template*, *keypoints_template*, *scores_template* and *descriptors_frame*, *keypoints_frame*, *scores_frame*. These outputs are fed to the *superglue_torchscript.pt* model, which matches the keypoints and returns the indices0, indices1, mscores0, mscores1 tensors. The tensors indices0, indices1 are the indices of the corresponding key points on the second image; ’-1’ returns if there is no match. Mscores0, mscores1—score to match a pair of points.

Launching the *tracker_init.pt*, *tracker_update.pt* models, which directly track the object.

## Results

351 images were used to test the model to determine the type of household appliances, and 370 images were used to test the model to determine the model of household appliances. Accuracy was chosen as a metric—the division of the ground truth into the total number of test samples (1). The test results of the classification models are given in Table 4.

1 $$AccuracyScore(y,\hat{y}) = \frac{1}{{n_{samples} }}\sum\limits_{i = 0}^{{n_{samples - 1} }} {1(\hat{y}_{1} = y_{i} )}$$

**Table 4:** Classification testing.

Model	Accuracy score
Type of household appliances	0.95
Model of household appliances	0.91

In order to ensure general validity of the classification results, five-fold cross-validation was performed.

To test the module, a prepared dataset of videos with labeled objects for tracking is required. Since at the time of the development of the module prototype, a sufficient test set of video data was not collected, the modeling and testing of the algorithm were carried out on open video sets. The success scores metric is chosen as the quality assessment metric, which is defined as the percentage of frames in which the predicted and reliable overlaps have an intersection area larger than a certain threshold (1). The test result is presented in Table 5.

2 $$SuccessScore(s,\hat{s}) = \sum\limits_{i = 0}^{{n_{samples} - 1}} {1(|\hat{s}_{i} - s_{i} | < threshold)}$$

**Table 5:** Tracking module testing results.

Dataset	Success scores
OTB-2015	0.680
LaSOT	0.511

For testing, an open dataset was used—OTB-2015 (Object Tracking Benchmark) dataset^31^, which is one of the classic tests for the object tracking problem, provides a good test for any family of trackers. The set contains 100 videos to evaluate the performance of the tracker. The success score is 0.680. A similar result was obtained on the LaSOT (Large-Scale Dataset for Object Tracking) dataset^32^, which contains a large number of video sequences (280 video sequences were used). The result of the evaluation (success scores) is 0.511 (Table 6).

**Table 6:** Mobile-based models for performance testing.

Model number	Description
1–2	Models without optimizations frame
3	Mobile_optimizer library function from PyTorch
4–6	Mobile_optimizer library function from PyTorch after merging the Convolution layers, BathNorm and Relu
7–8	Models with NNAPI PyTorch
9–11	A post-training static quantization

As mentioned above, the speed of tracking in a mobile application is important. Testing was carried out for 11 tracking models. Models 1 and 2 are simply traced and scripted models with no optimizations. Model number 3 is obtained with the mobile_optimizer library function from PyTorch. Models 4, 5 and 6 are also using the library function, but only after merging the Convolution layers, BathNorm and ReLU. Models 7 and 8 were prepared using computer vision model to use Android’s Neural Networks API (NNAPI) PyTorch. NNAPI provides access to powerful and efficient computational cores on many modern Android devices. In model 8, the last channel tensors are ordered using channel_last function from the PyTorch library. Models 9–11 have been quantized.

**Figure 10:**
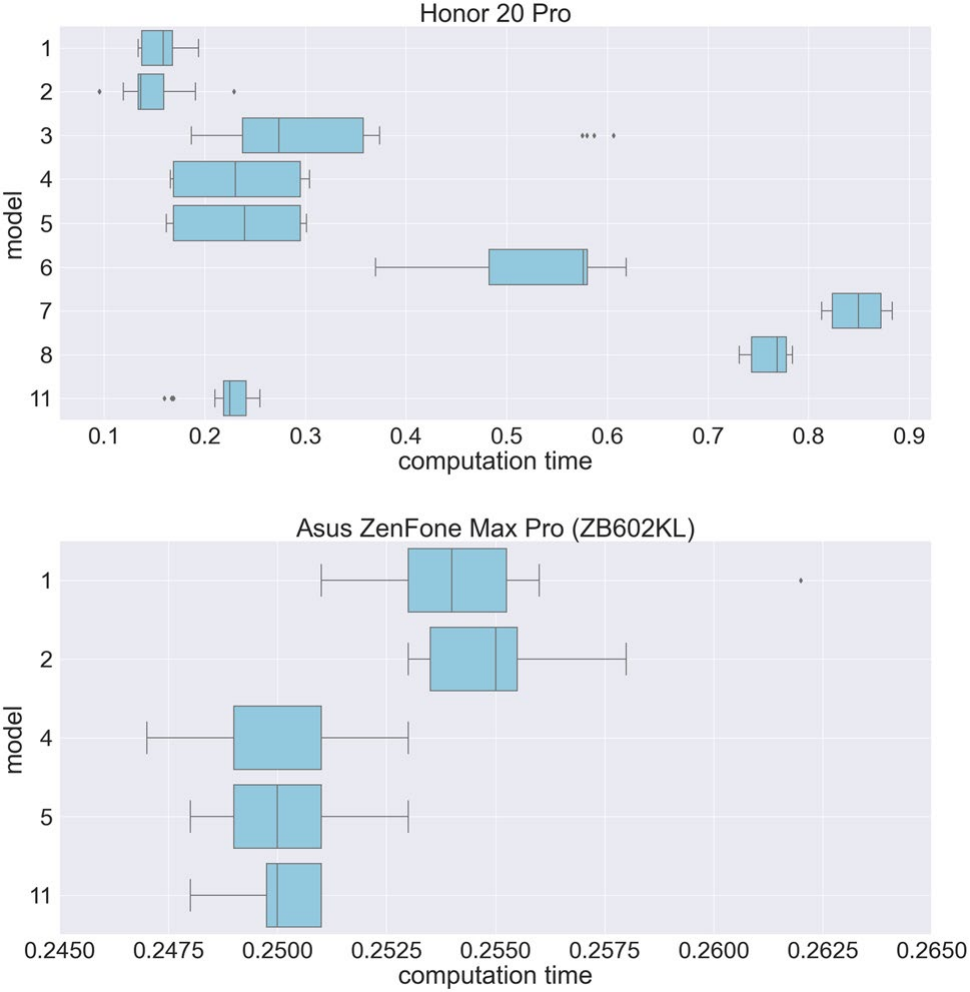
Statistics of tracking computational performance on the Android mobile platform.

Figure 10 shows the results of the performance tests for Honor 20 Pro and Asus ZenFone Max Pro (ZB602KL) phones based on the Android platform using boxplots for better visualization. Inference of the same image has been performed 10 times for each model. To eliminate a memory warm-up effect, the test was performed twice with direct and reversed model order. Similar experiments were carried out for the iOS platform. The part of the models cannot be evaluated on the Asus ZenFone Max Pro due to the lack of hardware support.

It can be seen that quantization became the most effective method, but the effect was not great. The alleged reason is that the tests were carried out on relatively old phones.

## Discussion

Thus, the mobile-based intelligent module was developed for the identification and tracking of household appliances. This module will be used for the augmented reality system that will help users speed up their familiarity with the functionality of household appliances. This task has been very actual, obviously, during the last decade because household appliances and industry equipment are becoming more and more complex. There are many industrial solutions that present augmented reality-based systems^13,14^. Smart home systems usually also include similar modules^33^.

Some solutions were described in the papers. The paper^16^ describes the GuideMe mobile application, which provides users with interactive instructions for home appliances based on augmented reality but it doesn’t present any intelligent modules with AI-based operations like classification or model recognition. The paper^17^ also solves similar problems, but the authors present their solution only for washing machines. Moreover, the proposed algorithm requires the knowledge of a 3D model of the object, which strongly differs from our solution.

During the development of the solution architecture and mobile applications, we encountered the following difficulties:

- There is a need to find a trade-off between the quality of the neural network-based models and their computational complexity, since the resources of mobile devices are very limited.
- A small amount of data. The resource intensity of working with raw data. Preparation of data for the neural network.
- Development was carried out during a period of frequent release of updates to the macOS and iOS operating systems that affect the functionality used, so there were problems with application builds and grids.
- Incomplete support for all functions in Python libraries for mobile devices.

The results evaluated using metrics show that the quality of the proposed solution is not inferior to state-of-the-art solutions. However, hardware and software are developing very quickly, new, more productive models of mobile devices are appearing, which can improve the implementation of neural network-based algorithms. At the same time, new neural network architectures have recently appeared, such as transformers, which can improve the quality of the models.

## Conclusions

We concluded that, since the resources of mobile devices are very limited, finding a trade-off between the quality of the neural network-based model implemented on the mobile device and its computational complexity are a challenging task. In particular, the difficulty of solving this problem is amplified for applications such as tracking. So, we present a space tracking algorithm for accurate positioning of visual objects relative to real world objects that were effectively implemented on different mobile devices with the use of optimization and quantization techniques.

Future work will be devoted to some research directions. First, a solution will be developed to quickly and easily add new models of household appliances with good quality in terms of metrics. Second, the work related to reducing computational complexity without losing quality of the presented tracking algorithm will continue.

## Data Availability

The datasets used during the current study are available from the corresponding author on reasonable request.
